# Spatio-temporal localization of *LlBOP* following early events of floral abscission in yellow lupine

**DOI:** 10.1007/s00709-019-01365-3

**Published:** 2019-04-16

**Authors:** Agata Kućko, Dariusz Smoliński, Emilia Wilmowicz, Aleksandra Florkiewicz, Juan de Dios Alché

**Affiliations:** 1 Chair of Plant Physiology and Biotechnology, Nicolaus Copernicus University, 1 Lwowska Street, 87-100 Toruń, Poland; 2 Department of Plant Physiology, Warsaw University of Life Sciences-SGGW (WULS-SGGW), Nowoursynowska 166 Street, 02-787 Warsaw, Poland; 3 Department of Cell Biology, Nicolaus Copernicus University, 1 Lwowska Street, 87-100 Toruń, Poland; 4 Centre for Modern Interdisciplinary Technologies, Nicolaus Copernicus University, 4 Wileńska Street, 87-100 Toruń, Poland; 5 Department of Biochemistry, Cell and Molecular Biology of Plants, Estación Experimental del Zaidín, CSIC, 1 Profesor Albareda Street, 18008 Granada, Spain

**Keywords:** Abscission zone, *BLADE ON PETIOLE*, Poly(A) mRNA, Reactive oxidative species

## Abstract

The phenomenon of excessive flower abscission in yellow lupine is a process of substantial interest to the agricultural industries, because it substantially affects the yield. The aim of this work was to provide an analysis of the changes taking place precisely in the abscission zone (AZ) during early stages of flower separation. We put particular emphasis on mRNA accumulation of *BOP* (*BLADE ON PETIOLE*) gene encoding a transcriptional factor so far considered to be essential for AZ formation. Our results show that the AZ displays a particular transcriptional network active in the specific stages of its function, as reflected by the expression profile of *LlBOP*. Noteworthy, spatio-temporal *LlBOP* transcript accumulation in the elements of pedicel vascular tissue reveals divergent regulatory mechanism of its activity. We have also found that AZ cells accumulate reactive oxidative species following abscission and what is more, become active due to the increasing amount of uridine-rich small nuclear RNA, accompanied by poly(A) mRNA intensive synthesis. Our paper is a novel report for *BOP* involvement in the AZ functioning in relation to the whole transcriptional activity of AZ and overall discussed regarding *BOP* role as a potential mobile key regulator of abscission.

**Electronic supplementary material:**

The online version of this article (10.1007/s00709-019-01365-3) contains supplementary material, which is available to authorized users.

## Introduction

The current state of knowledge on organ abscission in plants excludes the functioning of a single, universal mechanism for all species. However, based on previous experimental results, the separation process can be divided into four major steps: (1) the formation of anatomically distinct cells in the place of organ detachment, called abscission zone (AZ); (2) the acquisition by the AZ of competence to respond on signals that can stimulate abscission, such as phytohormones; (3) the activation of AZ followed by enzymatic dissolution of the middle lamellae catalyzed by cell wall remodeling enzymes; and, finally, (4) the formation of a protective layer (Patterson 2001). Much of our knowledge on the genetic and molecular basis of abscission processes derives mainly from studies of the model plant *Arabidopsis*. In agricultural applications, generative organ abscission directly affects crop yields, but so far, the knowledge concerning that aspect in crop species still remains poorly understood (Gil-Amado and Gomez-Jimenez 2013; Wang et al. 2013; Ying et al. 2016). However, recent experiments performed in *Arabidopsis*, tobacco (*Nicotiana tabacum*), as well as in a few species of legumes, has provided insight into the regulatory role of BLADE ON PETIOLE (BOPs) transcription factors in the formation of AZ (McKim et al. 2008;

Wu et al. 2012; Frankowski et al. 2015a; Couzigou et al. 2016). BOPs have been described as a part of the NPR1 (NON-EXPRESSOR OF PATHOGENESIS-RELATED GENES1) protein family, localized in the cytoplasm or the nucleus and regulated by intracellular redox balance controlling oligomer-to-monomer exchange under rapid oxidative burst condition (Ha et al. 2004). In a pioneer study, McKim et al. (2008) reported that *bop1bop2* double mutation results in a complete loss of floral organ abscission due to the absence of the distinct cellular anatomy typical of the AZ in *Arabidopsis*. However, the data regarding how the *BOP* genes function in the subsequent stages of this phenomenon have been not published to date.

Nowadays, scientific advances made especially on agriculturally important plants may provide candidate genes for control yielding and for improvement of breeding and harvesting practices. Among the legume (*Fabaceae*) family, yellow lupine (*Lupinus luteus* L.) is a highly significant crop, commercially cultivated in Eastern Europe, Southern Africa, Australia, and many Mediterranean countries. This species is characterized by high nutritional value and functional properties, e.g., the ability to fulfill nitrogen fixation, which is a sustainable alternative to chemical fertilizers. It also shows relatively high tolerance to abiotic stresses in comparison to other legumes and elevated content of high-quality proteins—a source for food and feed (Fernández-Pascual et al. 2007; Coba de la Peña and Pueyo 2012; Lucas et al. 2015). Excessive abscission of yellow lupine flowers is a limiting factor for productivity and, as a consequence, induces falling interest rates of cultivators. On the other hand, it represents an excellent experimental model to examine this phenomenon under laboratory conditions (van Steveninck 1958). Despite the great economic potential of yellow lupine, still little is known about the physiological and molecular basis of flower abscission in this plant. Our initial results showed that detachment of lupine flowers occurs in the AZ positioned at a predetermined site, located at the boundary between the flower and stem (Frankowski et al. 2015a). We have also found that during the differentiation of floral AZ, the expression of newly identified *LlBOP* was correlated with the changes at the cellular level (Frankowski et al. 2015a, 2015b).

It has been experimentally established that AZ formation is not sufficient for the execution of the separation processes. Generally, activation and the coordinated action of genes associated with endocytosis, vacuolar trafficking, cell wall modification, and those encoding the enzymatic components of the ROS-scavenging pathways inevitably lead to organ separation (Liu et al. 2013; Niederhuth et al. 2013; Liao et al. 2016).

It is well-known that during plant growth, the cells of the AZ become competent to respond on various stimuli, e.g., phytohormones that can induce separation events, consequently leading to a breakdown in cell adhesion (Taylor and Whitelaw 2001; Estornell et al. 2013). More recently, we have reported that abscisic acid (ABA) promotes the biosynthesis of ethylene (ET)—pointed out as a prominent stimulator of flower abscission in yellow lupine (Wilmowicz et al. 2016). Most interesting, both phytohormones caused transient accumulation of *LlBOP* transcripts (Frankowski et al. 2015a). Therefore, based on these observations, here we hypothesize that *LlBOP* may be involved not only in the early steps of abscission but also might be relevant in the further AZ activation. During the natural abscission of yellow lupine flowers, the pedicels above AZ become yellowish, whereas the stem fragment below remains green (Wilmowicz et al. 2016). Nevertheless, the procedure of artificial AZ activation by flower removal, leading to pedicel abscission has been commonly used in this research area and is a suitable tool to follow the biochemical and molecular changes during early stages of abscission (Roberts et al. 1984; Meir et al. 2010; Bar-Dror et al. 2011; Wilmowicz et al. 2016).

In this paper, we applied in situ hybridization methods to precisely localize *LlBOP* transcripts in AZ cells following its activation. The second objective of our research was the qPCR analysis of the *LlBOP* expression. New evidence is reported on the involvement of *LlBOP* in the functioning of floral AZ in yellow lupine by analyzing the spatio-temporal accumulation of transcripts, consistent with the appearance of ROS. What is more, we detected the U2 snRNA spliceosome component and the total poly(A) and used them as markers of mRNAs maturity, helping us to gain a comprehensive understanding of the transcriptional machinery involved, and the molecule organization during abscission processes.

## Material and methods

### Plant material and AZ activation procedures

The *Lupinus luteus* L. cv. Taper was selected for the experiments. Seeds provided by Poznan Plant Breeding (Tulce, Wiatrowo, Poland) were inoculated and sown following Frankowski et al. (2015b). Then, plants were cultivated in a growth chamber at a temperature of 22 ± 1 °C under long day conditions (110 μmol m^−2^ s^−1^, cool white fluorescent tubes by Polam, Warsaw, Poland).

We performed the experiments examining the transcriptional activity and the localization of *LlBOP* transcripts following artificial AZ activation. For this purpose, individual flowers were removed from plants by using a sharp razor blade above the AZ, leaving the pedicels, which induced their abscission (activated AZ). Subsequently, the tissue fragments containing AZs (excised 1 mm above and 1 mm below AZ) were excised from the plants after 2, 4, 6, 8, and 16 h (Supplementary Fig. 1c–g). The control sections (inactive AZ) were collected from green pedicels of flowers that showed no signs of abscission (Supplementary Fig. 1a). Parts of AZ flowers were harvested when features of natural abscission occurred, e.g., pedicels become yellowish and flowers withered (Supplementary Fig. 1b).

Plant material for all expression procedures was frozen in liquid nitrogen and stored at − 80 °C until RNA isolation, whereas tissue fragments for microscopy experiments were processed freshly. All experiments were designed in three independent biological replications.

### RT-qPCR

Frozen tissue samples were powdered in liquid nitrogen using a chilled mortar with pestle, and next, total RNA was extracted according to the manufacturer’s guidelines (NucleoSpin RNA Plant, MACHEREY-NAGEL GmbH & Co. KG, Düren, Germany). One microgram of isolated RNA was reverse transcribed with the Transcriptor First Strand cDNA Synthesis Kit (ROCHE Diagnostics GmbH, Germany) using anchored-oligo(dT)_18_ primers. Quantitative analyses were performed with the LightCycler Real-Time PCR Systems (ROCHE Diagnostics GmbH, Germany) using LightCyclerTaqMan Master (ROCHE Diagnostics GmbH, Germany). Amplifications of *LlBOP* (GenBank acc. no. KC792647) and *LlACT* (as an internal standard for normalization of expression value; GenBank acc. no. KP257588) from cDNA pools were performed under the same, optimized conditions as presented previously by Frankowski et al. (2015b) and using specific primers and hydrolysis probes listed in Supplementary Table 1. Reaction efficiencies (> 99%) were calculated based on the standard curves from serial dilutions of cDNA templates and relative expression values were calculated by LightCycler Software 4.1 (ROCHE Diagnostics GmbH, Germany). All experiments were carried out in three replicates with non-template control. The statistical analyses and graphical presentation of the obtained data were performed by the SigmaPlot 12 software. The data were presented as means ± standard deviation (SD) of three biological repeats. Student’s *t* test was used to calculate the significant differences compared with the control.

### Microscopy sample preparation

Freshly collected tissue fragments containing AZs from selected variants were immediately fixed in 4% paraformaldehyde (Fluka) and 0.2% glutaraldehyde (Sigma-Aldrich) in phosphate-buffered saline (PBS) buffer (pH 7.2) overnight at 4 °C. The material was dehydrated in increasing concentrations of ethanol-water series (10, 20, 30, 50, 75, 98, and 100%) containing 10 mM dithiothreitol (Sigma-Aldrich), subsequently supersaturated, and embedded in BMM resin (butyl methacrylate, methyl methacrylate, 0.5% benzoin ethyl ether, 10 mM dithiothreitol from Sigma-Aldrich). Polymerization was performed at − 20 °C under UV light. Semithin sections (1 μm) were cut with a glass knife on an Ultracut microtome (Reichert-Jung, Germany) and next were placed on glass slides covered with Biobond (BBInternational, UK). The BMM resin was removed by washing the slides in pure acetone, water, and PBS buffer.

### Probes for fluorescent in situ hybridization (FISH)

Two types of DNA probes were used. The first type of probes was directly labeled using Cy3 or Cy5 fluorophores for direct in situ hybridization as used in a previous FISH analysis (Smoliński and Kołowerzo 2012). The antisense DNA oligonucleotides (Genomed, Warsaw, Poland, and Sigma Proligo, USA) were used in the reactions of the detection of poly(A) RNA and U2 snRNA (Supplementary Table 2).

The second type of probes dedicated to *L. luteus* Blade-On-Petiole (*LlBOP*) mRNA (GenBank: KC792647.1) were designed using the Primer3 and RNA fold software available on http://frodo.wi.mit.edu/primer3/and http://univie.ac.at/cgi-bin/RNAfold.cgi websites.

Six of such probes were prepared with one residue of digoxigenin at the 5′-end (Genomed, Warsaw, Poland; Supplementary Table 2), then labeled at the 3′-end with digoxigenin nucleotides in a tailing reaction by terminal deoxynucleotidyl transferase (TdT) (Roche, Basel, Switzerland).

### Hybridization protocol

Before hybridization, the probes (Cy- and Dig-labeled) were resuspended in hybridization buffer (30% formamide (*v*/*v*), 4× SSC, 5× Denhardt’s buffer, 1 mM EDTA, and 50 mM phosphate buffer) to a concentration of 50 pmol. In situ hybridization was performed overnight at 28 °C as described previously (Smoliński et al. 2007). After 4× SSC, 2× SSC, and 1× SSC washing, nonspecific antigens were blocked with PBS buffer containing 0.1% acetylated BSA for 30 min in a humidified chamber. Next, all digoxigenin-labeled probes were detected using primary rabbit anti-DIG antibody (Life Technologies, USA) in 0.05% acetylated BSA in PBS (diluted 1:100) in a humidified chamber overnight at + 11 °C. The slides were subsequently washed with PBS buffer and incubated for 30 min in PBS buffer containing 0.01% acetylated BSA for the blocking of nonspecific antigens. Next, the material was incubated with the HRP-labeled goat anti-rabbit secondary antibody (Life Technologies, USA) in 0.05% acetylated BSA in PBS (diluted 1:1000) in a humidified chamber for 1 h at + 36 °C. The reaction was visualized with the use of tyramide, which was conjugated with Alexa 488 in 0.0015% H_2_O_2_ (Life Technologies, USA, diluted 1:200), and incubated at RT in a humidified chamber for 10 min according to Hyjek et al. (2015) and Life Technologies MP 20911 protocol. Finally, the slides were stained using Hoechst 33342 1 μg/ml for 3 min) to identify all nuclei and mounted in ProLong Gold antifade reagent (Life Technologies, USA). For the negative control, sense-labeled probes were used. In addition, antisense probes and ribonuclease-treated samples were used. All control reactions produced negative or negligible results compared with those of standard reactions (Supplementary Fig. 2).

### Microscopy

The results were registered with a Leica SP8 confocal microscope using a diode laser emitting light at a wavelength of 405 nm (UV excitation and Hoechst blue fluorescence), an argon-ion laser emitting light at a wavelength of 488 nm (blue excitation and Alexa 488 green fluorescence), a He-neon laser emitting light at a wavelength of 561 nm (green excitation and Cy3 yellow fluorescence), and a He-neon laser emitting light at a wavelength of 633 nm (red excitation and Cy5 red fluorescence). For confocal microscopy, long exposure time (100 Hz) and 40× (numerical aperture, 1.3) Plan Apochromat DIC H oil immersion lens were used. To minimize bleed-through between fluorescence channels, we employed low laser power (1–3% of maximum power), spectral detection, and sequential single-channel collection. For bleed-through analysis and control experiments, the Leica SP8 software was used.

### In situ ROS detection

In order to detect ROS, fresh samples of AZs were excised and incubated for 15 min in 50 μM DCFH_2_-DA (2′,7′-dichlorodihydrofluorescein diacetate) fluorochrome (Calbiochem, Darmstadt, Germany) prepared in MES solution (2-[N-morpholino]ethanesulfonic acid)-KCl buffer (5 μM KCl, 50 μM CaCl_2_, 10 mM MES (pH 6.15)) (Gomes et al. 2005). The negative control was treated with MES-KCl buffer only instead of fluorochrome. Observations were carried out using an epifluorescence stereomicroscope (M165FC) (Leica Microsystems GmbH, Germany) with 488 nm excitation, whereas images were gathered with digital camera controlled by the LAS Leica imaging software (Leica Microsystems, Bensheim, Germany).

## Results

### Quantification of *LlBOP* expression in the artificially induced AZs

**Fig. 1:**
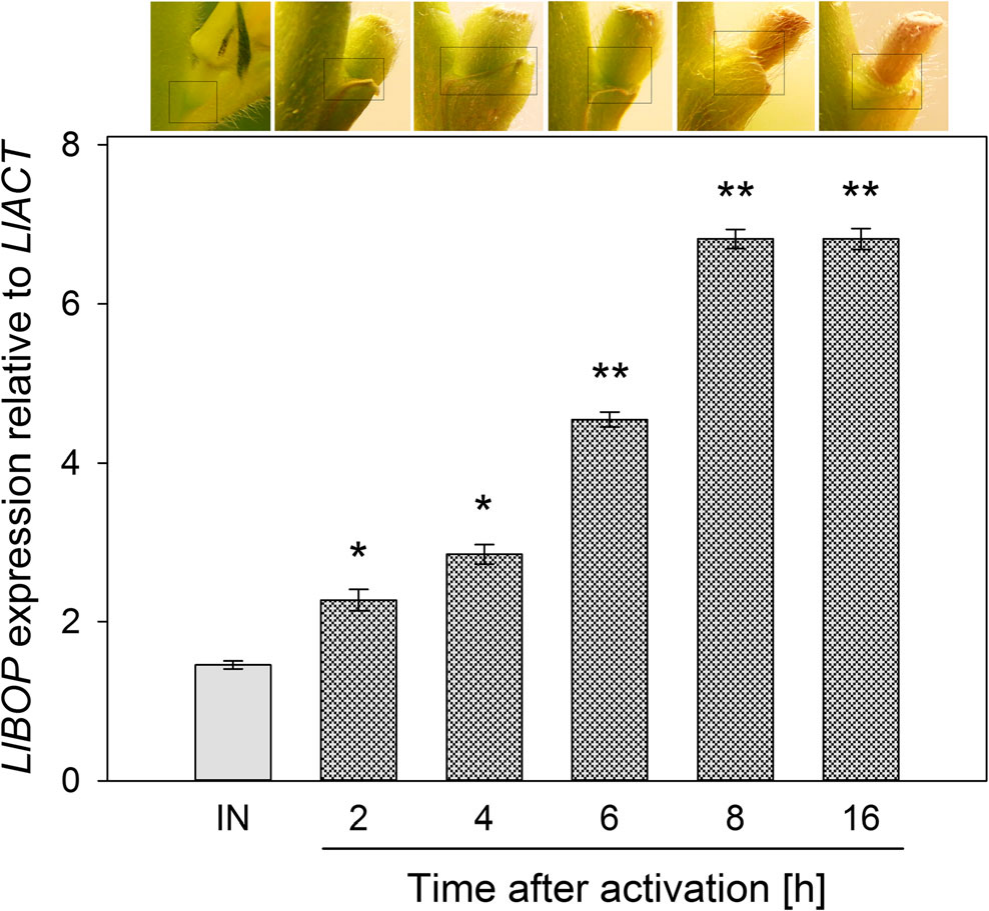
Transcriptional activity of *LlBOP* following artificial activation of flower abscission zone in yellow lupine. Total RNA was extracted from abscission zone (AZ) segments from the base of flowers at stage 5 of development (inactive AZ, IN). Additionally, AZ was artificially activated by organ removal and proper tissue fragments (~ 2 mm, shown within boxes in the photos) were sampled after 2, 4, 6, 8, and 16 h. *LlBOP* rapidly accumulated following artificial activation of AZ. The highest and similar transcript level at 8 and 16 h was observed. The *LlACT* gene was used to normalize the data, which are shown as averages of three technical replicates ± SE. Three independent RNA samples were used in the experiments. Significant differences to artificially activated AZ in comparison to inactive AZ (control) are indicated as **P < 0.01 and *P < 0.05

To determine the time of *LlBOP* activity, we examined the distribution of its transcript accumulation in the floral AZ after artificial activation. Following flower removal, the pedicels became yellow and the concomitant changes were easily observable (Supplementary Fig. 1c–g). The relative expression of the *LlBOP* rapidly upregulated during the hours subsequent to the artificial activation of AZ (Fig. 1). The examined gene accumulated at the highest and similar level at 8 and 16 h after induction of the separation processes. This value was almost five times higher than in the inactive (IN) AZ (control) (Fig. 1).

### *LlBOP* transcripts localization in inactive AZ

**Fig. 2:**
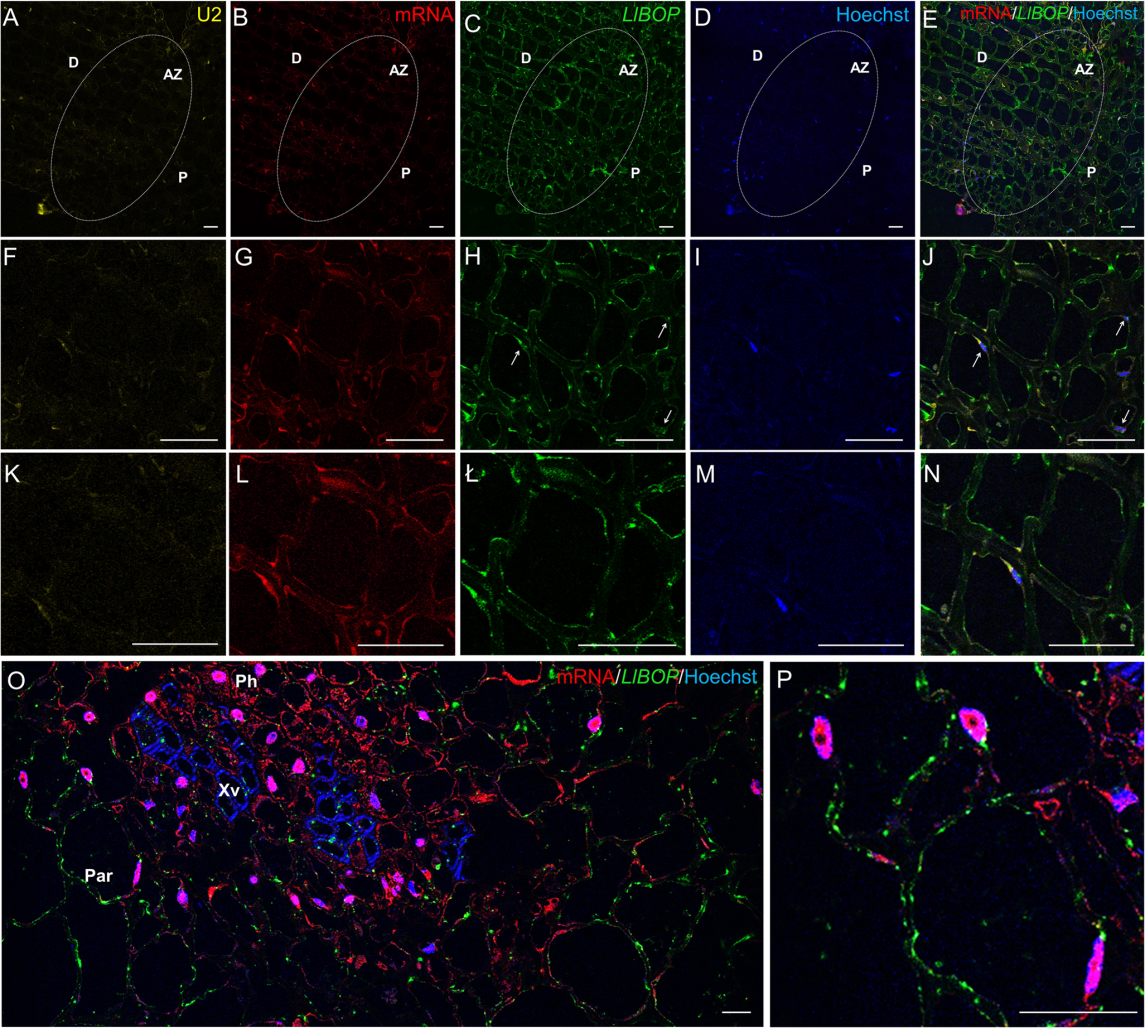
The expression of U2 snRNA (**a**, **f**, **k**), poly(A) mRNA (**b**, **g**, **l**), and *LlBOP* (**c**, **h**, **ł**) in the inactive floral abscission zone (AZ) (stage 5) and in the pedicel (**o**, **p**) determined by in situ hybridization in yellow lupine. Nuclei were stained with Hoechst 33342 (**d**, **i**, **m**). Merge of signals from immunostaining with poly(A) mRNA and *LlBOP* probes and Hoechst fluorescence (**e**, **j**, **n**, **o**, **p**). Higher magnification of the AZ region is shown in **f**–**j** and **k**–**n** (bottom row). The arrows indicate colocalization of *LlBOP* transcript with nuclei (**h**, **j**, **ł**, **n**). Poly(A) mRNA was detected in the nuclei (**g**, **i**, **l**, **m**) and a thin layer of cytoplasm (**g**, **l**). AZ cells are almost completely devoid of U2 snRNA. *LlBOP* was observed in the xylem vessels and in the cytoplasm of parenchyma cells within floral pedicel (**o**). More than half of nuclei contained poly(A) mRNA (**o**). AZ, abscission zone; D, distal side; P, proximal side; Par, parenchyma; Ph, phloem strands; Xv, xylem vessels. Scale bars, 40 μm

To further verify *LlBOP* involvement in the AZ functioning, studies concerning the localization of their transcripts were performed. In situ hybridization analysis showed a fluorescence signal of *LlBOP* expression in the thin layer of cytoplasm and nuclei of inactive AZ cells (Fig. 2c–j, ł, n), which partially colocalized with poly(A) RNA (Fig. 2b–l). On the contrary, U2 snRNA was almost not detectable in the AZ area (Fig. 2a–k). We observed a signal indicating *LlBOP* transcripts presence in the xylem vessels and parenchyma cells in comparison to phloem strands within floral pedicels (Fig. 2o, p).

### *LlBOP* transcripts localization after artificial AZ activation

**Fig. 3:**
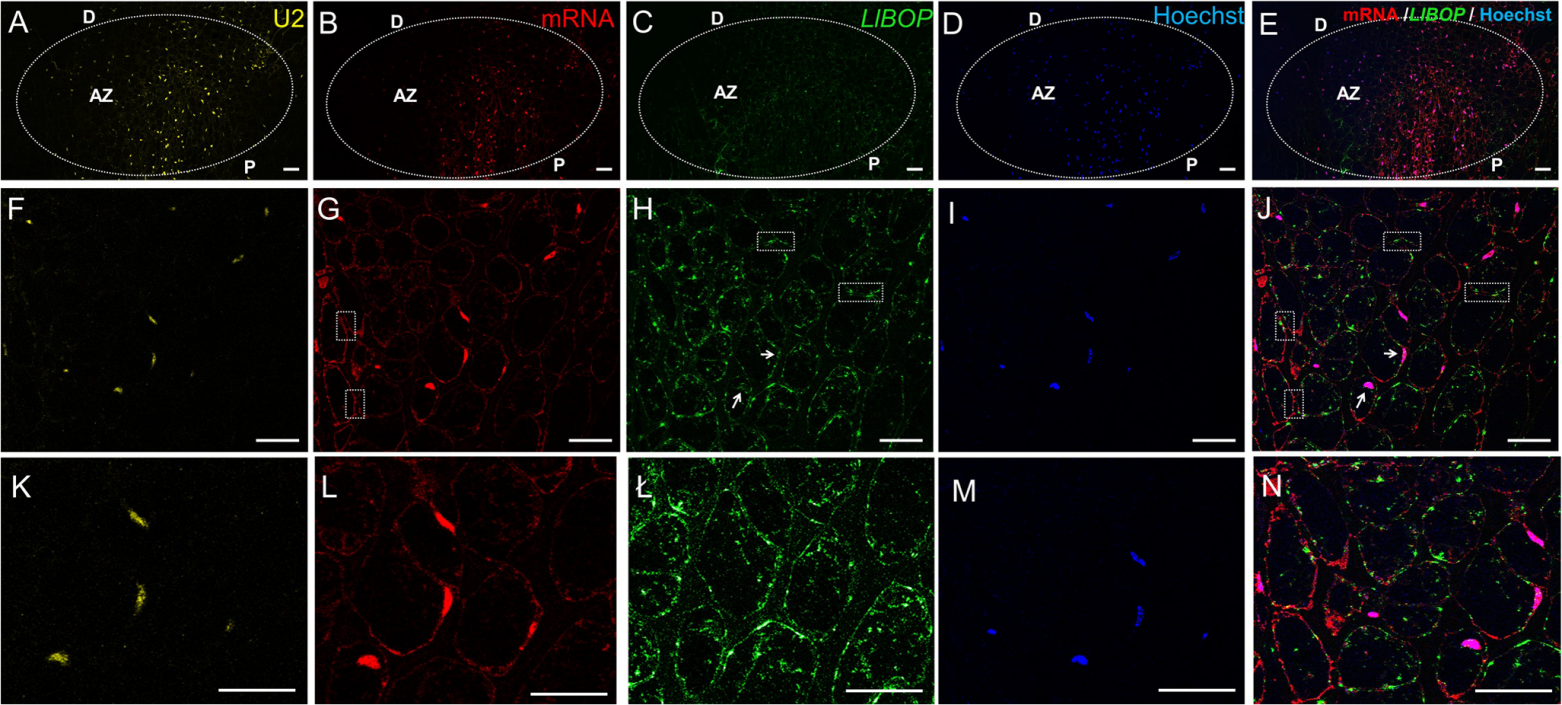
Distribution of U2 snRNA (**a**, **f**, **k**), poly(A) mRNA (**b**, **g**, **l**), and *LlBOP* (**c**, **h**, **ł**) in the flower abscission zone (AZ) of yellow lupine 2 h after its artificial activation. Hoechst 33342 staining of nuclei (**d**, **i**, **m**). Merge of poly(A) mRNA and *LlBOP* fluorescent signal with additional Hoechst 33342 staining (**e**, **j**, **n**). Higher magnification of the AZ region is shown in **f**–**j** (bottom row). The *LlBOP* transcript strongly accumulated in the cytoplasm, as well as in the nuclei indicated by arrows (**h**, **j**, **ł**, **n**). U2 snRNA signal was restricted to the nuclei (**f**, **k**). Poly(A) mRNA was concentrated in the cytoplasm and nuclei (**g**, **l**). White boxes indicate the *LlBOP* and poly(A) mRNA staining in the intracellular spaces (**g**, **h**, **j**). AZ, abscission zone; D, distal side; P, proximal side. Scale bars, 40 μm

The abundance of *LlBOP* transcripts in the AZ increased 2 h after activation (Fig. 3c–j, ł, n) when compared with control (IN AZ) (Fig. 2c, h, ł). At the cellular level, the cytoplasm was dotted with *LlBOP* transcripts, but single stained aggregates were found also in the nuclei (Fig. 3h–n). Particularly interesting was the presence of some punctate signals of red and green fluorescence indicating poly(A) mRNA (Fig. 3g) and *LlBOP* transcript (Fig. 3h) presence, in the intercellular spaces, visible at the higher magnification as structures connecting cells. A similar pattern of U2 snRNA (Fig. 3f, k) and poly(A) mRNA (Fig. 3g, l) staining in the whole, big nuclei (Fig. 3i, m), especially from cells within the central region of the pedicel was observed (Fig. 3a, b). However, poly(A) mRNA probe was also concentrated in the cytoplasm (Fig. 3g, l), often in the area of *LlBOP* presence (Fig. 3j, n).

**Fig. 4:**
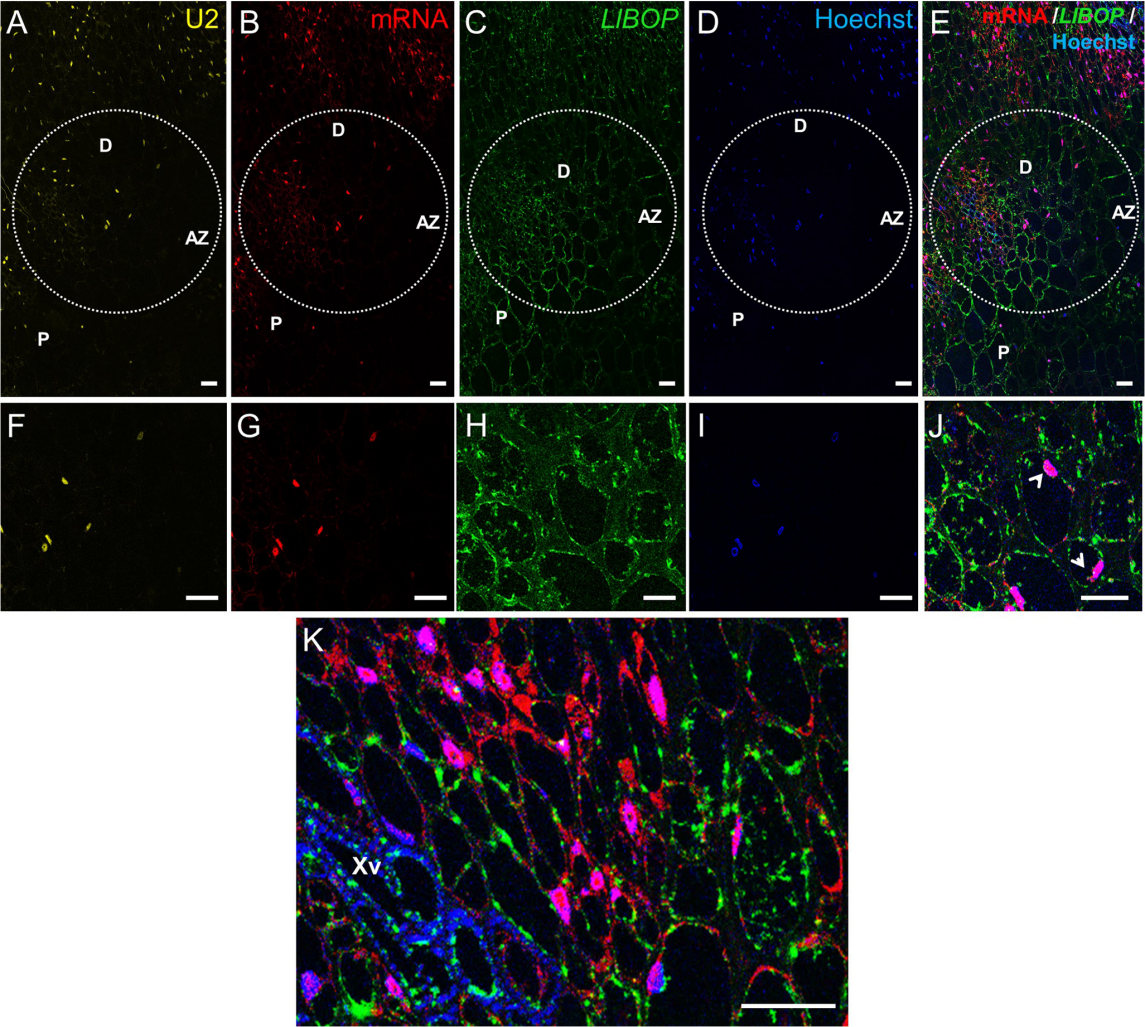
Detection of U2 snRNA (**a**, **f**), poly(A) mRNA (**b**, **g**), and *LlBOP* (**c**, **h**) in the flower abscission zone (AZ) and in the pedicel’s vascular tissue (**k**) 8 h after artificial activation in yellow lupine. DNA staining performed using Hoechst 33342 (**d**, **i**). Poly(A) mRNA, *LlBOP* and Hoechst fluorescence after merging (**e**, **j**, **k**). Merged images of **b**, **c**, and **d** (**e**). AZ area is shown in higher magnification (**f**–**j**) (bottom row). *LlBOP* transcript in the cytoplasm and nuclei (arrows) of AZ cells was detected (**h**, **j**). Merged signals of poly(A) mRNA, *LlBOP*, and Hoechst in the floral pedicels (**k**). Green fluorescence was clearly visible in the xylem vessels and neighboring cells (**k**). mRNA accumulated in the cytoplasm and nuclei (**k**). AZ, abscission zone; D, distal side; P, proximal side; Xv, xylem vessels. Scale bars, 40 μm

Next, we have found that 8 h after artificial AZ activation, U2 snRNA (Fig. 4a, f) and poly(A) mRNA (Fig. 4b, g) were limited to the nuclei in both cases. The simultaneous signal of poly(A) mRNA and Hoechst 33342 staining in the AZ cells resulted in a pink fluorescence after merging (Fig. 4j). The *LlBOP* transcripts in the cytoplasm and nuclei of AZ cells were detected (Fig. 4h, j). What is more, the vascular elements and neighboring cells in the pedicels were intensely stained with *LlBOP* probe (Fig. 4k), in a similar way to AZ (Fig. 4j).

### *LlBOP* transcript accumulation in the naturally active AZ

**Fig. 5:**
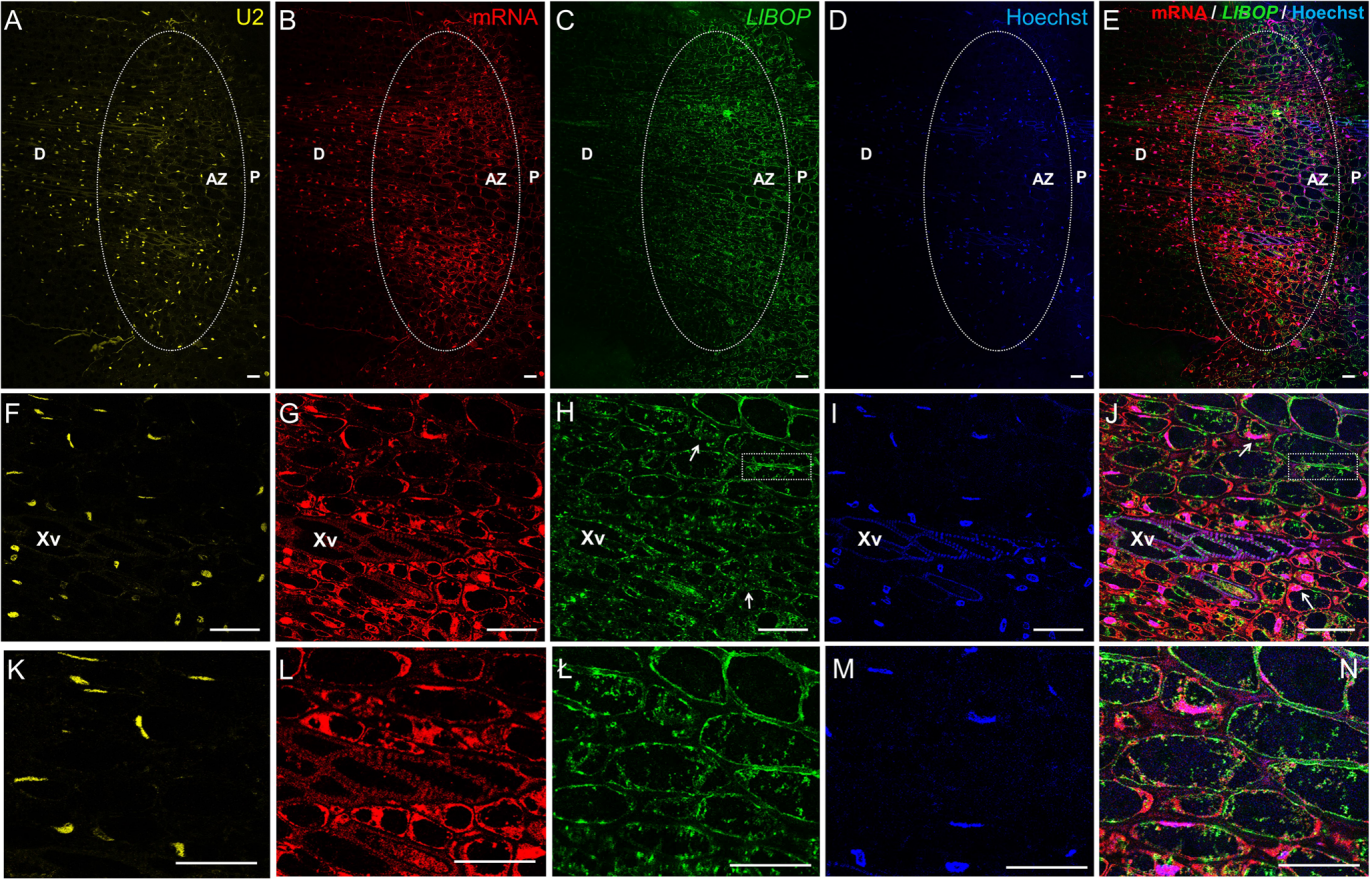
The expression of U2 snRNA (**a**, **f**, **k**), poly(A) mRNA (**b**, **g**, **l**), and *LlBOP* (**c**, **h**, **ł**) in the abscission zone (AZ) prepared from naturally abscised flowers of yellow lupine. Hoechst 33342 staining of nuclei (**d**, **i**, **m**). Merge of signals from immunostaining with poly(A) mRNA and *LlBOP* probes and Hoechst 33342 fluorescence (**e**, **j**, **n**). Bottom row (**f**–**j**, **k**–**n**) represents an enlarged region of AZ cells. The strong increase in the all examined molecules in the AZ cells and xylem vessels was observed. Detection of *LlBOP* in the numerous nuclei and in the plasmodesmata is indicated by arrows and white boxes, respectively (**h**, **j**, **ł**, **n**). *LlBOP* also accumulated in the cytoplasm (**h**, **j**, **ł**, **n**). U2 snRNA was limited to the nuclei (**f**, **k**). Labeling from poly(A) mRNA in the nuclei and cytoplasm was visible (**g**, **l**). AZ, abscission zone; D, distal side; P, proximal side; Xv, xylem vessels. Scale bars, 40 μm

Finally, we performed in situ hybridization in tissue sections from AZ segments of the base of flowers in which abscission was in process. The cells of the naturally active AZ contain U2 snRNA (Fig. 5a), poly(A) mRNA (Fig. 5b), and *LlBOP* transcripts (Fig. 5c), which were more in comparison to AZ cells prior to abscission (Fig. 2), or the artificially activated AZ cells (Figs. 3 and 4). Furthermore, after staining with Hoechst 33342, the active AZ was easily distinguishable, because of the presence of numerous strongly labeled nuclei (Fig. 5d), in which a pink signal indicating the presence of poly(A) mRNA was detected after merging (Fig. 5). A considerably larger amount of poly(A) RNA was present in the nuclei compared to the cytoplasm (Fig. 5g–n). Both compartments of the cells adjacent to vascular bundles were significantly enriched in poly(A) mRNA (Fig. 5g, l), which partially colocalized with the dispersed signal of *LlBOP* (Fig. 5h, j, ł, n). Moreover, the *LlBOP* transcripts occurred in the xylem vessels (Fig. 5h, j). At the cellular level, green fluorescence in the periphery of the cell, even in the extracellular spaces, was detected (Fig. 5h, j). In turn, U2 snRNA (Fig. 5a–k) and Hoechst 33342 (Fig. 5d–m) staining revealed that nuclei contain large amounts of that spliceosome component.

### Fluorescence detection of ROS in the AZ and surrounding tissues

**Fig. 6:**
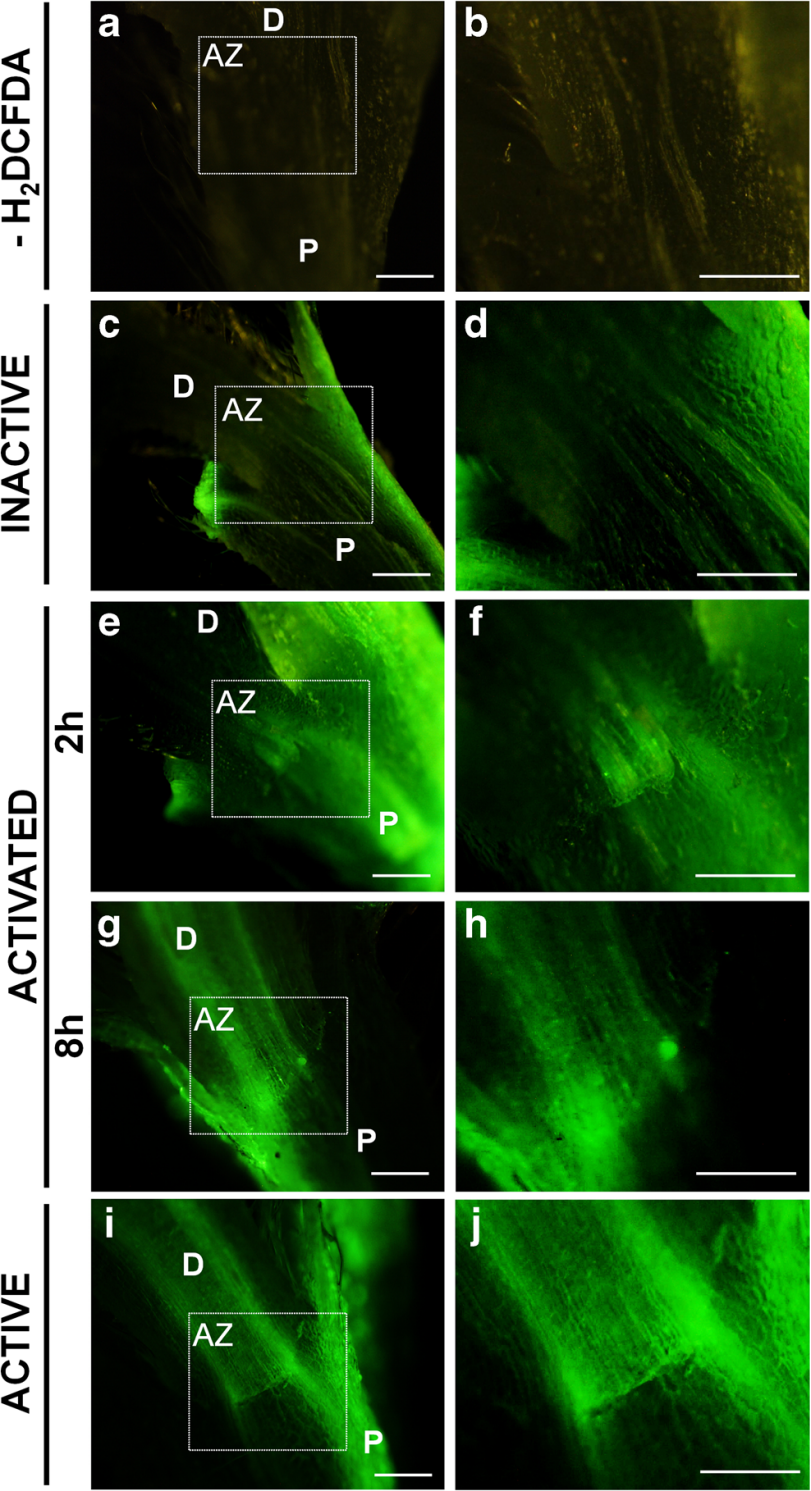
Detection of ROS with H2DCFDA in the floral abscission zone (AZ) of Lupinus luteus. No signal van be seen for flower not treated with H2DCFDA (**a**, **b**). The presence of ROS after fluorochrome application is shown by green fluorescence. AZ area of inactive control is unlabeled (shown within white boxes - **c**, **d**). Two hours after artificial activation signal was observed at the base of flower pedicels (**e**, **f**) and subsequently stretched to the vascular bundles (8 h) (**g**, **h**). The whole AZ area and pedicel’s vascular bundles of the naturally abscised flower were labeled (**i**, **j**). AZ – abscission zone, D – distal side, P – proximal side. Scale bars: 2 mm.

To examine whether ROS synthesis accompanied abscission, staining of flower pedicel was performed (Fig. 6). Before abscission (IN AZ), the green fluorescence, indicating ROS presence, was limited to the peripheral region of the sections the fracture of excision and the surface of small, neighboring leaf AZ (Fig. 6c), but was almost undetectable in the floral AZ area (Fig. 6d). After treatment with DCFH_2_-DA fluorochrome, the signal was observed initially (2 h after activation) in the center of AZ (Fig. 6e, f) and subsequently (8 h after activation) stretched to the pedicel’s vascular tissue (Fig. 6g, h). We detected strong labeling across vascular bundles, both at the distal and proximal side of naturally active AZ, but more intense in the pedicel (Fig. 6i, j). No fluorescence, either over the background or the control experiments, was observed in the absence of the DCFH_2_-DA fluorochrome treatment (Fig. 6a, b).

## Discussion

*BLADE ON PETIOLE* genes encoding transcription factors BOPs are members of a small gene family in *Arabidopsis* that have been extensively studied in recent years, which resulted in an increasing number of publications on their involvement in the control of many aspects of plant growth and development, including leaf patterning, inflorescence and flower development, nodulation, as well as organ separation (Hepworth et al. 2005; Khan et al. 2014). Considerable progress in the regulation of the AZ formation mediated by BOPs has been made during the recent years. So far, researchers put emphasis on the essential role of BOP in the AZ differentiation; however, most aspects concerning its contribution in the AZ functioning have been yet to experimentally verified. McKim et al. (2008) have not ruled out that BOP controls subsequent stages leading to abscission. Therefore, here, to get a first insight into the whole transcriptional activity and *LlBOP* influence in the recent events of abscission, we determined the changes in the expression, localization of *LlBOP* accompanying by U2 snRNA and poly(A) mRNA transcript accumulation in the floral AZ cells after abscission-promoting treatment in yellow lupine. Generally, it seems that the qPCR studies (Fig. 1) are compatible with the dynamic changes in the cellular localization of *LlBOP* transcript during AZ activation (Figs. 3 and 4). The *LlBOP* mRNA was presented in the nucleus and cytoplasm (Fig. 2). We observed *LlBOP* transcript in the AZ cells after artificial activation (Figs. 3 and 4) and AZ of naturally abscised flowers (Fig. 5). It strongly supports the thesis about *LlBOP* involvement in the separation processes, not only in the early events but also at the final stages. Based on the changing pattern of *LlBOP* localization in the floral AZ and pedicel’s vascular bundles of yellow lupine (Fig. 2; Fig. 4; Fig. 5), it is tempting to speculate that examined mRNA can be synthesized in vascular elements or/and be transported from other places to AZ cells. The presence of a fluorescence signal at the borders of the AZ cells connecting them across the cell wall indicates that *LlBOP* local translocation may be mediated by formed plasmodesmata, clearly visible already 2 h after activation (Fig. 3h, j) and even in the naturally active AZ (Fig. 5h, j, ł, n). During organ separation extensive membrane trafficking associated with high occurrence of branched plasmodesmata serving as channels for the lytic enzymes, secretion was observed within AZ cells in tomato (*Solanum lycopersicum*) and *Elaeis guineensis* (Bar-Dror et al. 2011; Roongsattham et al. 2012). However, it cannot be excluded that *LlBOP* transcript appearance in the vascular tissue is connected with BOP involvement in proper vasculature development as proposed in *Arabidopsis thaliana* (Ha et al. 2003).

In yellow lupine, an expression of U2 snRNA spliceosome component and total poly(A) mRNA in the AZ cells when abscission was initiated (Fig. 3f–l) and, especially abscission in the process (Fig. 5f–l) implicates the link between separation and total transcriptional activity of AZ cells. Such a localization pattern proved that the newly synthesized transcripts appeared in the nucleus just after transcription (Figs. 3g, l and 4g) and following abscission demonstrated also cytoplasmic localization (Fig. 5g, l)—a place of potential translation. Our data reflect transcriptionally active sites with high metabolic activity of AZ cells and are in line with findings performed in other species, e.g., soybean (*Glycine max*), olive (*Olea europea*), and *Rosa chinensis* which revealed increased expression of AZ-specific genes, encoding, inter alia, cell wall modifying enzymes and many pathogenesis-related (PR) genes, transcription factors, and vesicle trafficking components involving small GTPases (Gil-Amado and Gomez-Jimenez 2013; Gao et al. 2016; Kim et al. 2016). The colocalization of U2 snRNA and poly(A) mRNA (Figs. 3f–l; 4f, g; and 5f–l) in the floral AZ of yellow lupine indicates that newly transcribed genes undergo maturation in spliceosomes. The protein products of these molecules are probably essential for cell wall hydrolysis and modifications, e.g., cellulases, polygalacturonases, and may directly influence the middle lamella dissolution followed by cell wall rapture, as was confirmed in other plant species (Bonghi et al. 1992; Kalaitzis et al. 1997; Belfield et al. 2005; Ogawa et al. 2009). The evidence supporting the thesis concerning de novo protein synthesis during separation event initiation was provided on the basis of studies conducted in *Oenanthe stolonifera* (Eo and Lee 2011). Similar cytosol localization pattern of *LlBOP* and poly(A) mRNA (Figs. 3g–l, ł and 5g–l, ł) suggests that newly formed *LlBOP* transcript is processed into mature mRNA and may be translated in this compartment. LlBOP contains BTB/POZ (Broad-Complex, Tramtrack, and Bric-a-brac/Pox virus and Zinc finger) evolutionarily conserved domain (Dong 2004; Frankowski et al. 2015a). We revealed also the presence of characteristic motifs, along with Cys residue within an N-terminal domain BTB/POZ essential for redox control in the LlBOP predicted protein sequence (Frankowski et al. 2015a). It confirms the functioning of signaling mechanism conserved with NPR1 proteins, which have been shown to express under oxidative stress conditions (Dong 2004; Hepworth et al. 2005; Norberg et al. 2005). In this paper, we clearly demonstrate the similar pattern of *LlBOP* and ROS localization during AZ functioning. ROS presented initially, in the AZ area (2 h) and vascular tissue (8 h) after abscission induction (Figs. 3h, j, ł, n ; 4k; and 6e–h). Subsequently, when abscission in the process, ROS were detected at the base of flower pedicel and across vascular bundles (Fig. 6i, j), similarly to *LlBOP* (Fig. 5c, e). These results may suggest a functional relationship and a possible link between *LlBOP* expression as a consequence of the intensive production of ROS—the key contributors to altered cell redox potential. In tomato and pepper (*Capsicum*) ROS are accumulated prior to and stimulate flower and leaf abscission (Sakamoto et al. 2008; Bar-Dror et al. 2011). Moreover, H_2_O_2_ induces expression of the cell wall-degrading enzyme at the execution phase of abscission (Sakamoto et al. 2008).

Collectively, our results providing a piece of critical experimental evidence supporting the hypothesis that *LlBOP* transcript is involved in the floral abscission in agronomically important plant—yellow lupine. What is more, the data presented here constitute, to the best of our knowledge, the first report regarding the *LlBOP* contribution in the AZ activation, leading to novel insights into the cellular and molecular mechanisms of separation processes. With using localization approaches, we also confirmed that AZ represents a particular transcriptional network active in the specific stages of its functioning, supported by the spatial and temporal appearance of spliceosomal machinery and newly transcripts, of which *LlBOP* plays a substantial role. Such data provide essential information for further mechanisms that regulate crop yields and for the elucidation of new potential molecular indicators to improve genetic breeding.

## Electronic supplementary material

ESM 1 (PPT 2413 kb)
