# Supplementary material for: Spatio-temporal localization of LlBOP following early events of floral abscission in yellow lupine
Source: Protoplasma. 2019 Apr 16;256(5):1173–83. doi: 10.1007/s00709-019-01365-3 (PMC6713700; doi:10.1007/s00709-019-01365-3)
Supplement: Supplementary file 1 — (PPT 2413 kb) [file 709_2019_1365_MOESM1_ESM.ppt]

## Slide 1
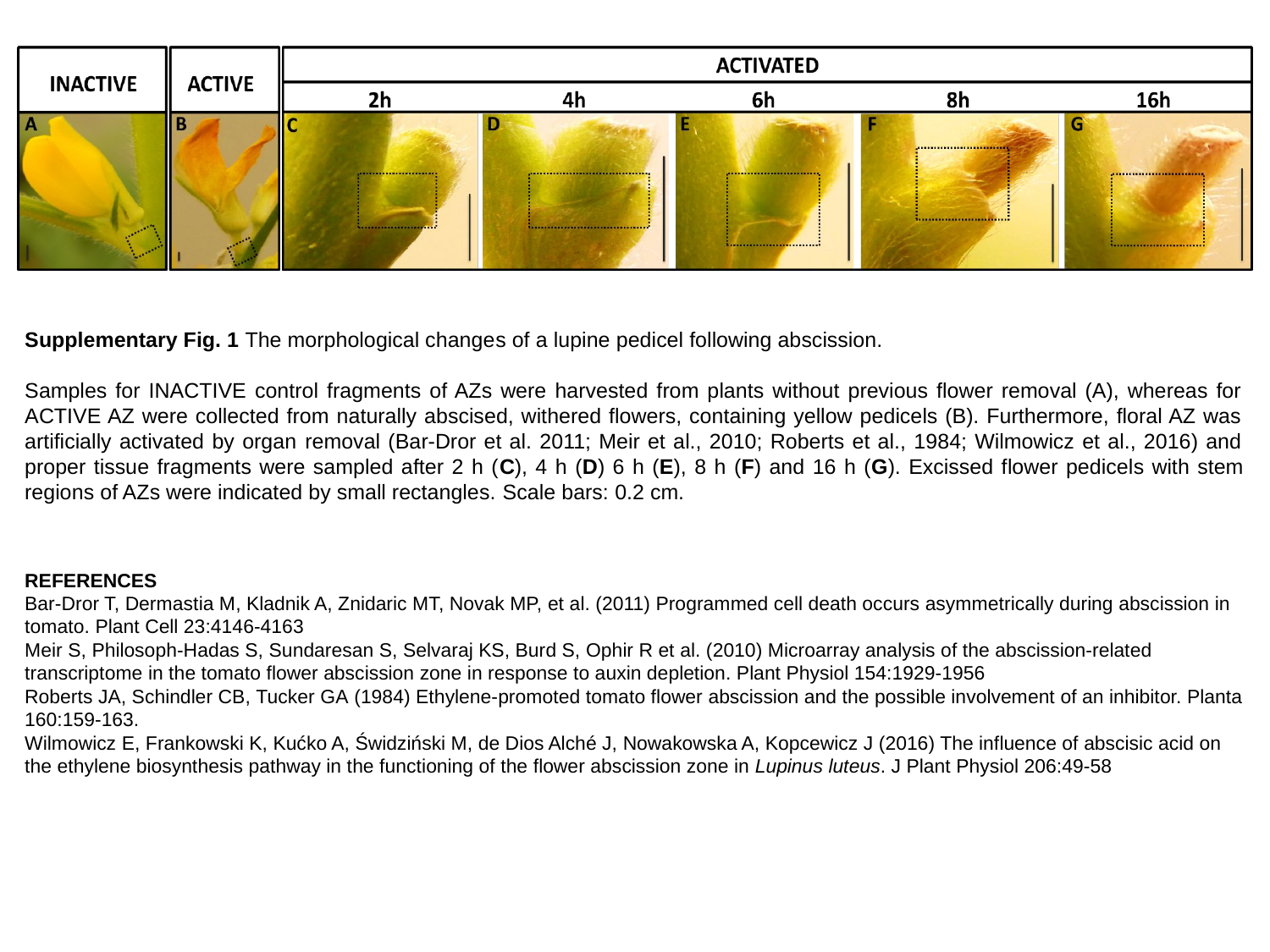

Supplementary Fig. 1 The morphological changes of a lupine pedicel following abscission.
Samples for INACTIVE control fragments of AZs were harvested from plants without previous flower removal (A), whereas for ACTIVE AZ were collected from naturally abscised, withered flowers, containing yellow pedicels (B). Furthermore, floral AZ was artificially activated by organ removal (Bar-Dror et al. 2011; Meir et al., 2010; Roberts et al., 1984; Wilmowicz et al., 2016) and proper tissue fragments were sampled after 2 h (C), 4 h (D) 6 h (E), 8 h (F) and 16 h (G). Excissed flower pedicels with stem regions of AZs were indicated by small rectangles. Scale bars: 0.2 cm.
REFERENCES
Bar-Dror T, Dermastia M, Kladnik A, Znidaric MT, Novak MP, et al. (2011) Programmed cell death occurs asymmetrically during abscission in tomato. Plant Cell 23:4146-4163
Meir S, Philosoph-Hadas S, Sundaresan S, Selvaraj KS, Burd S, Ophir R et al. (2010) Microarray analysis of the abscission-related transcriptome in the tomato flower abscission zone in response to auxin depletion. Plant Physiol 154:1929-1956
Roberts JA, Schindler CB, Tucker GA (1984) Ethylene-promoted tomato flower abscission and the possible involvement of an inhibitor. Planta 160:159-163.
Wilmowicz E, Frankowski K, Kućko A, Świdziński M, de Dios Alché J, Nowakowska A, Kopcewicz J (2016) The influence of abscisic acid on the ethylene biosynthesis pathway in the functioning of the flower abscission zone in Lupinus luteus. J Plant Physiol 206:49-58

## Slide 2
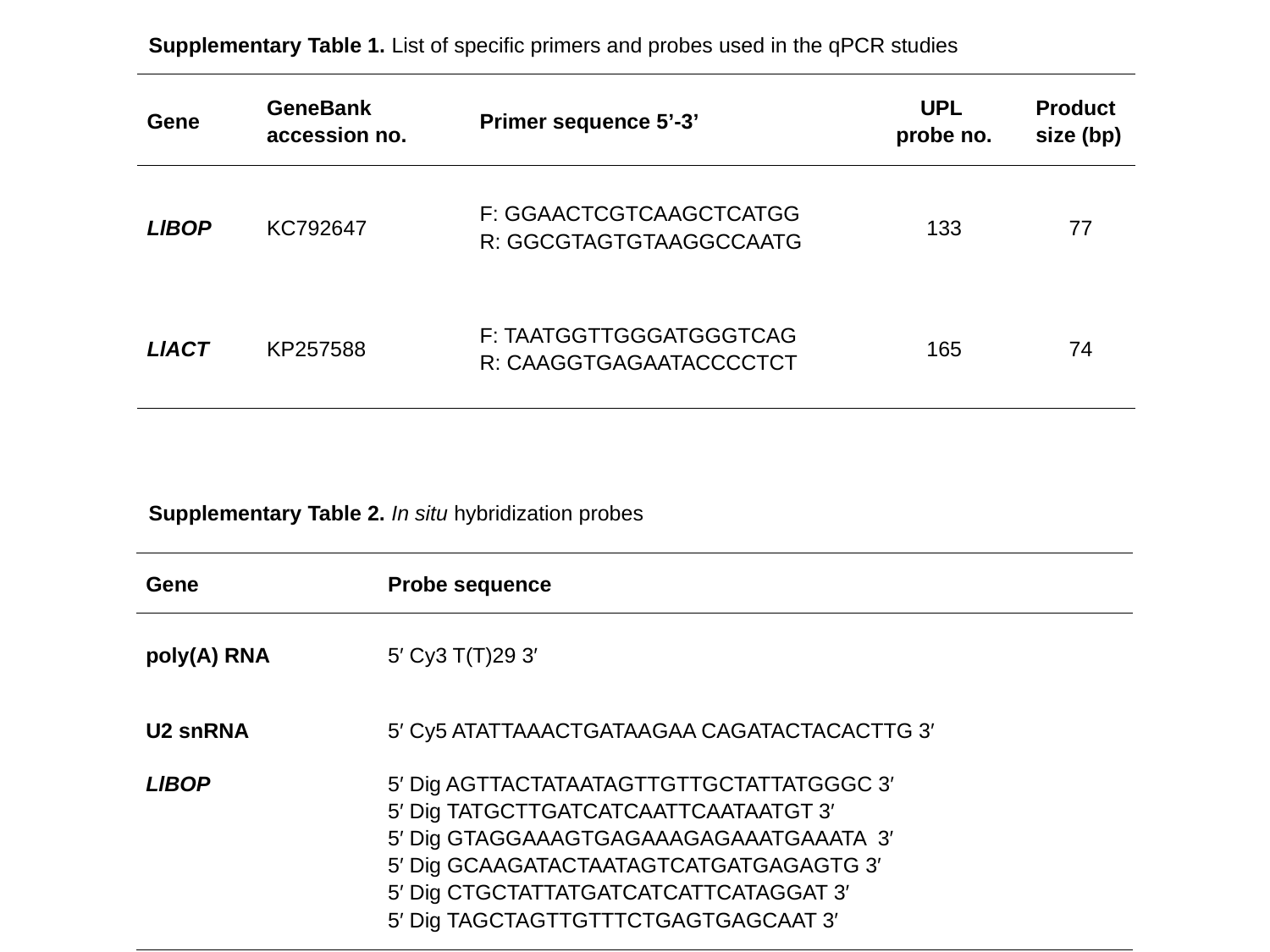

Supplementary Table 1. List of specific primers and probes used in the qPCR studies
| Gene | GeneBank accession no. | Primer sequence 5’-3’ | UPL probe no. | Product size (bp) |
| --- | --- | --- | --- | --- |
| LlBOP | KC792647 | F: GGAACTCGTCAAGCTCATGG R: GGCGTAGTGTAAGGCCAATG | 133 | 77 |
| LlACT | KP257588 | F: TAATGGTTGGGATGGGTCAG R: CAAGGTGAGAATACCCCTCT | 165 | 74 |
Supplementary Table 2. In situ hybridization probes
| Gene | Probe sequence |
| --- | --- |
| poly(A) RNA | 5′ Cy3 T(T)29 3′ |
| U2 snRNA | 5′ Cy5 ATATTAAACTGATAAGAA CAGATACTACACTTG 3′ |
| LlBOP | 5′ Dig AGTTACTATAATAGTTGTTGCTATTATGGGC 3′ 5′ Dig TATGCTTGATCATCAATTCAATAATGT 3′ 5′ Dig GTAGGAAAGTGAGAAAGAGAAATGAAATA 3′ 5′ Dig GCAAGATACTAATAGTCATGATGAGAGTG 3′ 5′ Dig CTGCTATTATGATCATCATTCATAGGAT 3′ 5′ Dig TAGCTAGTTGTTTCTGAGTGAGCAAT 3′ |

## Slide 3
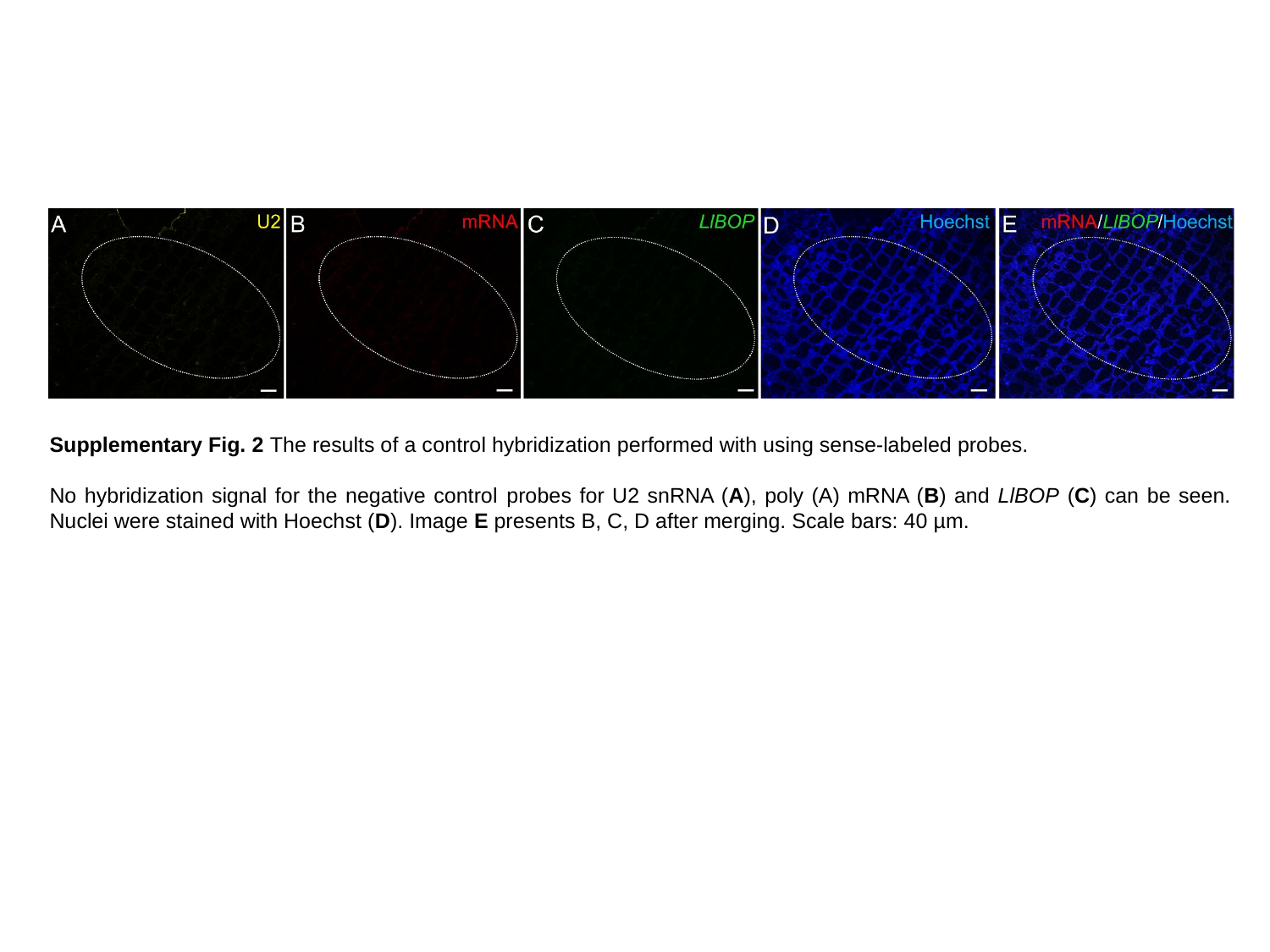

Supplementary Fig. 2 The results of a control hybridization performed with using sense-labeled probes.
No hybridization signal for the negative control probes for U2 snRNA (A), poly (A) mRNA (B) and LlBOP (C) can be seen. Nuclei were stained with Hoechst (D). Image E presents B, C, D after merging. Scale bars: 40 µm.
